# Abrasive Waterjet (AWJ) Forces—Potential Indicators of Machining Quality

**DOI:** 10.3390/ma14123309

**Published:** 2021-06-15

**Authors:** Libor M. Hlaváč, Massimiliano P.G. Annoni, Irena M. Hlaváčová, Francesco Arleo, Francesco Viganò, Adam Štefek

**Affiliations:** 1 Department of Physics, Faculty of Electrical Engineering and Computer Science, VSB–Technical University of Ostrava, 17. listopadu 2172/15, 70800 Ostrava-Poruba, Czech Republic; irena.hlavacova@vsb.cz (I.M.H.); adam.stefek.st@vsb.cz (A.Š.); 2 Department of Mechanical Engineering, Politecnico di Milano, Via La Masa 1, 20156 Milano, MI, Italy; massimiliano.annoni@polimi.it (M.P.G.A.); francesco.vigano@polimi.it (F.V.); 3 WatAJet S.r.l., Via Tomasetto 31, 21010 Besnate, VA, Italy; francesco.arleo@watajet.com

**Keywords:** abrasive waterjet, tangential force, normal force, kerf characteristics, cutting wear, deformation wear

## Abstract

The necessity of monitoring the abrasive waterjet (AWJ) processes increases with the spreading of this tool into the machining processes. The forces produced on the workpiece during the abrasive waterjet machining can yield some valuable information. Therefore, a special waterjet-force measuring device designed and produced in the past has been used for the presented research. It was tested during the AWJ cutting processes, because they are the most common and the best described up-to-date AWJ applications. Deep studies of both the cutting process and the respective force signals led to the decision that the most appropriate indication factor is the tangential-to-normal force ratio (TNR). Three theorems concerning the TNR were formulated and investigated. The first theorem states that the TNR strongly depends on the actual-to-limit traverse speed ratio. The second theorem claims that the TNR relates to the cutting-to-deformation wear ratio inside the kerf. The third theorem states that the TNR value changes when the cutting head and the respective jet axis are tilted so that a part of the jet velocity vector projects into the traverse speed direction. It is assumed that the cutting-to-deformation wear ratio increases in a certain range of tilting angles of the cutting head. This theorem is supported by measured data and can be utilized in practice for the development of a new method for the monitoring of the abrasive waterjet cutting operations. Comparing the tilted and the non-tilted jet, we detected the increase of the TNR average value from 1.28 ± 0.16 (determined for the declination angle 20° and the respective tilting angle 10°) up to 2.02 ± 0.25 (for the declination angle 30° and the respective tilting angle of 15°). This finding supports the previously predicted and published assumptions that the tilting of the cutting head enables an increase of the cutting wear mode inside the forming kerf, making the process more efficient.

## 1. Introduction

Investigations on the abrasive waterjet (AWJ) machining, both from the quantitative and the qualitative points of view, have been in progress for four decades [1,2]. The basic description of the AWJ cutting process was proposed by Hashish [3], who based his analyses on the previous works in the erosion investigations presented by Bitter [4,5], who described the deformation wear mode in abrasive processes, and Finnie [6], who focused on the cutting wear description. Nevertheless, the effort in improving the AWJ machining process understanding and description is still in a progress. This is due to the fact that no complex and all factors, parameters and variables, including descriptions and/or models, have been finished yet, since their preparation is very complicated. The contemporary models and descriptions can be divided, at least from the material behavior point of view, into a group focused on ductile materials [7,8], represented namely by aluminum and its alloys, copper and its alloys or mild steels, and a group aimed at brittle materials [9,10,11,12] including strong but brittle steels, ceramics, glass, rocks, etc. The attempts to prepare some linking theoretical description [13] and physical models unifying description of the curvature of the fluid jet trajectory in the kerf [14] and its impact on the product distortion [15] turn out to be not sufficiently beneficial to replace the regression based models or the descriptions being used in practice for years. Therefore, a further diffusion and improvement of the AWJ machining techniques is conditioned by investigating partial problems and deepening detailed knowledge of technology.

In the last two decades, the AWJ investigations have focused not only on the “traditional” cutting [16,17] but also on milling of aerospace materials [18,19,20,21], turning [22], precise piercing [23], drilling [24], grinding [25] and polishing of metallic glass [26] or the very hard materials [27]. The role of an abrasive material is also studied very often. Suitable properties characterizing mineral particles were sought [28], the role of the mixing process was investigated [29], the shape and hardness effects in milling processes were studied [30], the influence of recycling processes on abrasive material properties and their efficiency were investigated [31,32] and searching for the appropriate abrasive materials for deep cutting in the strong materials was performed [33]. The main phenomena having the substantial influence on a product shape were also investigated in many articles. The researchers studied changes in the kerf width [34], the cutting front profile [35] and the jet lag [36]. The important studies were aimed at negative phenomena influencing the product distortion, namely the trailback [15] and the taper [37,38]. Some ways to correct these cutting imperfections are presented in the recent works [39,40]. The typical striations produced on the cutting walls were investigated as a qualitative indicator [14] or a phenomenon influencing roughness of walls of the carved product [41]. Roughness of the kerf walls is the important characteristic of machined composites [42] or very strong and abrasive resistant materials [43]. All the published works show the necessity of introducing the new measuring techniques [44]. Some attempts to detect the machining quality through the acoustic emissions were published for milling process [45] or cutting [46]. The acquisition of cutting head vibrations is another technique applied for the AWJ machining monitoring [47,48,49]. Nevertheless, the results seem to be questionable, because many phenomena and factors are influencing the final detected acoustic and vibration signals. Some researchers even allege that more than 25% of information from such sources may be incorrect [50], because the typical frequencies of individual signals can be produced by other sources than the predicted ones. Moreover, the dimensions of the machined material, the dimensions of the machine, its stiffness and other factors substantially influence the signals, namely the eigen frequencies.

Therefore, the idea arose to measure the forces acting on samples with comparable dimensions, because the forces are directly related to the cutting mechanism itself. During cutting operations, the jet exerts a force on the workpiece consisting of three components. The tangential force, F_x_, acts parallel to the direction of the traverse speed; it is denoted as TF. It is assumed to correspond to the cutting force (CF). The lateral force, F_y_, acts perpendicular to the TF in the same plane; it is denoted as LF. The normal force, F_z_, perpendicular to F_x_ and F_y_, is denoted as NF, and it includes the deformation force (DF). The lateral force is not an important factor in the case of linear cuts, as its value is usually 50–100 times lower than the TF one. The idea of using the force measurement to monitor and control the AWJ machining processes arose several years ago. This was partly inspired by the research activities aimed at measuring the single-axis force generated by impacts of the pure water jets [51]. These measurements were used in particular to determine the jet diameter [52], the pulsation [53] and the jet breakup [54] of the pure water flows. Some basic relations between trends of the single-axis forces and the AWJ characteristics were presented in Reference [55]. Further investigation of the AWJ forces was focused on the mixing process [56], the AWJ diameter determination [57,58] or the recoil force of the hand tools [59].

However, the investigation of all three force components in the Cartesian coordinate system requires a special type of force sensor. Therefore, such a force sensor was designed, manufactured and patented by a research group at the VSB-Technical University of Ostrava (Ostrava, Czech Republic) in 2011 [60]. It is able to measure the *x*–*y*–*z* forces during the AWJ cutting process. Its current main use is focused on the research area where it helps to deepen the knowledge about the relationship between cutting forces and the properties of both the material and the jet, but its modification for use in the manufacturing process monitoring is possible. The sensor has been used for measuring many samples during several years. Some of the problems which may occur on some cutting devices were highlighted in the conference contribution [61]—detection of random strikes, DC voltage shifts or random electromagnetic pulses. The impact of the internal structure of steel materials on force measurements [62] and the relationship between the relative traverse speed and the cutting-to-deformation force ratio (CDFR) were also published [63]. However, all of those results were obtained on the cutting tables without any possibility of cutting-head tilting.

The present work summarizes the most important findings acquired on tables with the tilting cutting head. The TF and NF can be linked with the cutting and deformation wear of the material inside the kerf. Therefore, the corresponding forces are measured, and their ratio (TNR) is compared for several metallic materials considering the different declination angles and the jet-axis tilting angles to verify the theorems based on observations during experiments aimed at some hypotheses predicted from the theory. The theorems express the observed regularities that apply to the ratio of the tangential to normal forces (TNR) in the AWJ cutting processes. The results are sufficiently consistent to allow expectations that the force measurements can be used for the online monitoring of the AWJ machining processes and even the online process control (after the appropriate sensor modifications).

## 2. Theoretical Base

All theoretical calculations presented in this article are based on Hlaváč’s models. Therefore, the most important parts of this theoretical base are summarized here. The basic model presented in [13,15] is used namely for determination of the limit traverse speed marked $v_{Plim}$. The Equation (1) enables calculation of the limit traverse speed from the jet characteristics and respective material properties. The limit traverse speed ($v_{Plim}$) is the maximum $v_{P}$ dividing the material so that the bridges are not longer than 25% of the kerf width and are not closer than 50 mm to each other (see also the introduction of this parameter in [13] and the explanation in [14]). The respective jet velocity loss, $\alpha_{e}$, could be calculated from the jet and material properties (Equation (2)) and the jet characteristics, but usually not all necessary parameters are available. Therefore, the value of $\alpha_{e}$ is determined experimentally [64], namely due to the interaction time uncertainty. The respective equations for calculation of the limit traverse speed (1) and the jet velocity loss in the interaction process (2) are based on the jet and material characteristic parameters:

(1) $$v_{\mathrm{Plim}}={\left[\frac{C_{A}\;S_{p}\;\pi \;d_{o}\;\sqrt{2\rho_{j}p_{j}^{3}\;e^{-5\xi_{j}L}}\;(1\;-\;\alpha_{e}^{2})}{8\;H\;\left(p_{j}\rho_{m}\alpha_{e}^{2}\;e^{-2\xi_{j}L}\;+\;\sigma_{m}\rho_{j}\right)}\right]}^{\frac{2}{3}}\;-\;v_{\mathrm{Pmin}}$$

(2) $$\alpha_{e}=1-\frac{\sqrt{2p_{j}^{3}}\;HVt_{i}}{8\sqrt{\rho_{j}}\;\sigma_{m}a_{m}}$$

Nomenclature (basic SI units where available):

$v_{Plim}$  the limit traverse speed;
$C_{A}$  the coefficient modifying abrasive water jet performance according to the changing content of abrasive below “saturation level” (above this level, the jet performance increases no more and $C_{A}=1$);
$S_{P}$  ratio between the quantity of non-damaged grains (i.e., not containing defects) and the total quantity of grains in the supplied abrasive material;
$d_{o}$  diameter of the water nozzle;
$\rho_{j}$  density of abrasive jet (conversion to homogeneous liquid);
$p_{j}$  pressure obtained from Bernoulli’s equation for liquid with density and velocity of abrasive jet;
$\xi_{j}$  attenuation coefficient of abrasive jet in the environment between the focusing tube outlet and the material surface;
L  stand-off distance (distance between exit of the focusing tube and material surface);
$\alpha_{e}$  coefficient of abrasive water jet velocity loss in the interaction with material (experimentally determined);
H  material thickness;
$\rho_{m}$  density of material being machined;
$\sigma_{m}$  strength of material being machined;
$v_{Pmin}$  minimum limit traverse speed of cutting—correction for the zero traverse speed (usually $v_{Pmin}=a_{n}/60$ is used, where $a_{n}$ is the average abrasive particle size after the mixing process inside the mixing head and focusing tube);
HV  material hardness;
$t_{i}$  interaction time;
$a_{m}$  mean size of particles (elements) of material—grains or their chips.

The jet velocity loss ($\alpha_{e}$) is, in principle, valid only for parameters used in the value determining experiments. Changing of any parameter (or parameters) raises the need to re-perform the respective experiments required for determination of the respective quantity, namely the limit traverse speed. However, using the appropriate theoretical relationships it is possible to convert the required data valid for the different conditions to the ones for an actual state without necessity of the additional time-consuming experimental work. This was applied during the experimental part of work presented in this article. The experiments were performed on the materials studied in the past [14,15,39,64], several times, from the various points of view. Therefore, the respective limit traverse speeds for these samples were determined and are known for the parameters of the machine used in the Laboratory of Liquid Jet at the VŠB-Technical University of Ostrava (Ostrava, Czech Republic). Unfortunately, this machine has no possibility of the controlled cutting-head tilting. Therefore, to perform the research presented in this article, it was necessary to find some academic partner with the appropriate equipment. The best opportunity to carry out the intended research program was to make it a part of the international co-operation with the Politecnico di Milano (Milan, Italy). To avoid the time-consuming experiments aimed at determination of the limit traverse speed for different conditions, the recalculation of this key factor from the values determined in Ostrava to those valid in Milan was performed by using the equation prepared from Hlaváč’s theoretical base:

(3) $$v_{PlimM}=v_{PlimO}\frac{d_{oM}}{d_{oO}}$$

The variables $v_{PlimM}$ and $v_{PlimO}$ are the respective $v_{Plim}$ values determined and/or calculated for equipment in Milan and Ostrava; $d_{oM}$ and $d_{oO}$ are the respective diameters of water nozzles (orifices). Other constant parameters used in Milan and Ostrava are either identical or mutually compensated. This analysis is based on Hlaváč’s model, i.e., Equations (1) and (2). The respective limit traverse speeds calculated for experiments are presented in the chapter aimed at the methods and tools.

## 3. Methods and Tools

### 3.1. Measuring Device

**Figure 1:**
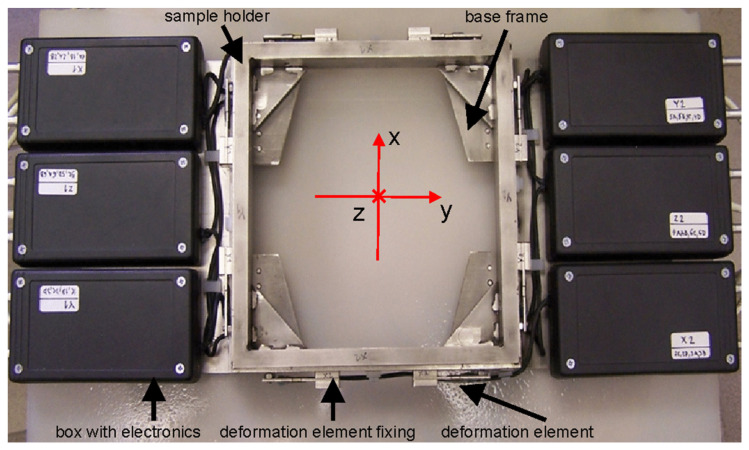
Force sensor view from the top (in the direction of the flowing jet from the non-tilted cutting head—*z*-axis).

The AWJ cutting forces are measured through a patented force sensor [60]. The whole device consists of the frame with deformation elements, the sample holder frame and the electronics separated into six plastic boxes. Each of these boxes includes the amplifier of the electric signals raised from the strain gauges in the respective direction. These gauges are pasted on the deformation elements mounted on the main measuring frame and touching the sample holder frame via pointing bits. The overall design is shown in Figure 1. The force-measuring device is located horizontally with respect to the Earth’s surface, and the AWJ is flowing vertically.

The first measurements were performed in Ostrava, as the part of the continuing research plan. Besides the fact that the equipment in the Laboratory of Liquid Jet at the VŠB-Technical University of Ostrava (Ostrava, Czech Republic) has no possibility of cutting head tilting, there is also a serious problem with a strong electromagnetic noise there. In spite of the effort of several firms and specialists, the noise cannot be eliminated sufficiently for the force measurements. Therefore, it was agreed with colleagues in Milan to perform the research program during teaching stays in laboratories of the Politecnico di Milano (Milan, Italy). During implementation of the entire common research plan (several years) the equipment of the laboratory in Milan changed. Therefore, two machines with almost identical settings were used for measurements there. Finally, results performed on both machines are presented. The older measurements were performed on AWJ machines Tecnocut IDRO 1740 (C.M.S. SpA, Zogno, Italy) and the most recent data were measured on Intermac Primus 322 Metal (INTERMAC, Pesaro, Italy). The base of the limit traverse speeds for a substantial part of tested materials was determined (as it was explained in Section 2) during former experiments presented in References [13,14,15] and performed on the machine PTV WJ 1020-1Z-EKO (PTV s.r.o., Hostivice, Czech Republic) used in the Laboratory of Liquid Jet at the VŠB-Technical University of Ostrava (Ostrava, Czech Republic). Therefore, the limit traverse speeds necessary for experimental work in Milan were recalculated for different conditions by using Equation (3). Thus, the time was saved that would otherwise be necessary for the time-consuming experiments for the limit traverse speeds determination.

**Figure 2:**
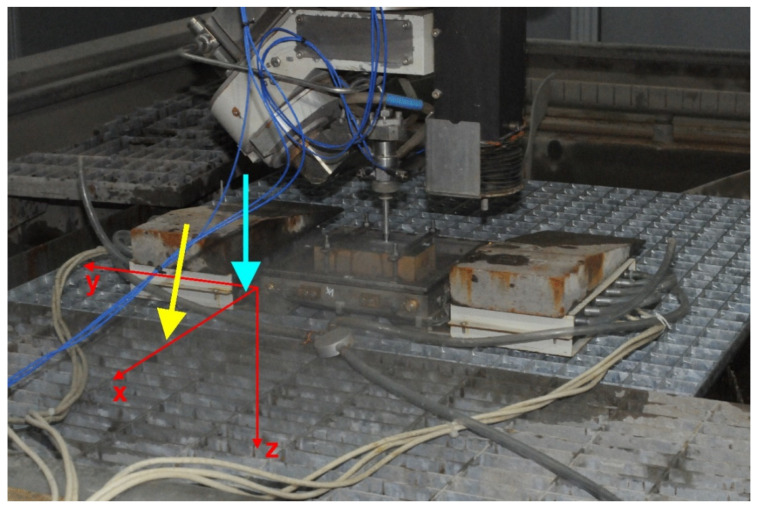
Detail of the force sensor on the cutting table in Milan. Heavy metal blocks are used to fix the device to the grid. The orthogonal axes system is sketched in the figure. Directions of the non-tilted and the tilted jets, both lying in the plane *x*–*z*, are indicated by the cyan and yellow arrows respectively. Actual position of the cutting head matches the non-tilted state.

Detail of the force sensor on the cutting table is presented in Figure 2. The electric parts of the device are protected by pressurized air blowing through the boxes containing the electronic circuits and then through the tube opened to the environment (at the closer side of the force measuring device in Figure 2).

### 3.2. Force Signals Acquisition and Processing

**Figure 3:**
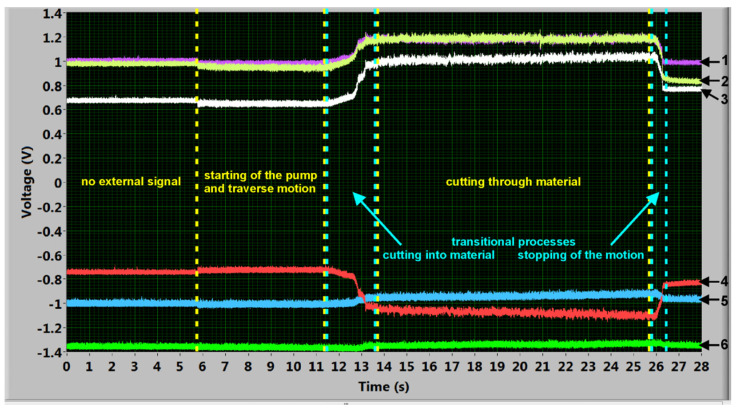
An example of force signal measurement with tilted cutting head: (1) one portion of NF signal, (2) second portion of NF signal, (3) TF signal positive, (4) TF signal negative, (5) LF signal positive and (6) LF signal negative.

The signals were measured using the software Signal Express 2012 and it is evaluated in software LabVIEW 12 (Version 12, 2012), both supplied by the National Instruments Corp., Austin, TX, USA). An example of the typical voltage signals measured with tilted cutting head is shown in Figure 3.

The most important signals in the presented investigation are those corresponding with the TF in the direction of the traverse speed and the NF ones. The NF signals are marked in plots as 1 and 2. Due to the sensor design the whole normal force signal is divided into two signals and the resulting NF is a sum of them. The TF signals are marked as 3 and 4 in Figure 3. The total signal is an average of the absolute values of the positive and negative signals in the *x*-axis. The signals representing LF (5 and 6) are not investigated during these experiments, because LF values are very small during linear cuts (as it is evident from the record presented in Figure 3) and so they are not significant for the presented research. The total LF signal (in the case of the linear cuts in *x*-direction like presented in this study) is an average of the absolute values of the positive and negative signals in the *y*-axis, similarly to the TF.

**Figure 4:**
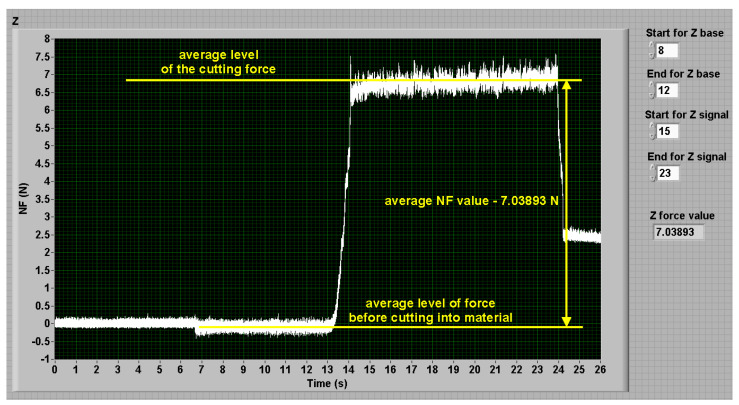
Determination of the NF force: it is the sum of the NF voltage signals (sum of yellow and magenta in Figure 3) recalculated through calibration equations.

The used processing starts by selecting of the respective parts of the signal for the force value determining. A part of the signal level with the switched pump and the traverse movement, but prior cutting into material, forms the base level (see the part “starting of the pump and traverse motion” in Figure 3). A part of the signal corresponding to the stable cutting through material (with the exception of the initial transitional process corresponding to the cutting into material and the final transitional process corresponding to the jet traverse movement stopping) forms the level of the respective force during machining (the part “cutting through material” in Figure 3). Both parts of the signal (selected for calculations) should be at least two seconds long. The respective average values of force levels are calculated from both parts of the signal (Figure 4) and their difference is considered to be the force value in the respective axis. The total NF value is the sum of partial signals 1 and 2 in Figure 3. The selection of the signal parts and respective calculation of the force values is a part of the signal processing programmed in the LabVIEW (Figure 4). The respective calibration equations were determined by using normalized weights. The respective equations are implemented into the LabVIEW programs for signal processing.

### 3.3. The Cutting Machines Used for Research

The three AWJ cutting machines were used in preparation of the present study, as it was explained in Section 2 and Section 3.1. The parameters of machines are summarized in Table 1.

The limit traverse speeds, $v_{Plim}$, necessary for setting the cutting conditions in the research plan in Milan were recalculated by using Equation (3) from the values determined in Ostrava. Both limit traverse speeds are summarized in Table 2 for a set of the tested metals. Other constant parameters used in Milan and Ostrava are either identical or mutually compensated, using an analysis based on Hlaváč’s model [13]. The cutting-capability decrease caused by the lower operating pump pressure and the higher stand-off distance in Milan (Table 1) are compensated by the higher water and abrasive mass flows according to the used model—Equations (1) and (2).

## 4. Experimental Plan and Results

The analyses of signals throughout the years show several interesting findings which form the base for the experimental research presented and discussed in this article:

**Theorem** **1.** *The tangential-to-normal force ratio (TF-to-NF ratio, i.e., TNR) is closely connected to the actual-to-limit traverse speed ratio (* $v_{P}$ *-to-* $v_{Plim}$ *ratio).*

This theorem is based on studies performed on the samples from the identical material with the different thickness. Previously, the relationship between the TNR and the $v_{P}$-to-$v_{Plim}$ ratio has been studied on steel samples with the identical elemental composition but the different strength characteristics caused by various thermal treatments to demonstrate the independence of the TNR behavior on metal composition and density [62]. This research is aimed at the influence of the sample thickness.

**Theorem** **2.** *Forces representing the interaction between the abrasive waterjet and the cut material indicate the prevailing type of wear during the measurement.*

This theorem claims that the TNR indicates the prevailing type of wear at a certain traverse speed, $v_{P}$. The growing $v_{P}$ should cause higher NF in the case of the normal jet impact. The deformation wear increases, while the cutting wear decreases, making it so that the cutting-to-deformation wear ratio (CDR) decreases, as well as the TNR, and it is smaller in the case of higher $v_{P}$ values. This relation should be valid for material thicknesses that allow the full development of both the cutting and the deformation wear (for the applied jet properties).

**Theorem** **3.** *The jet axis tilting towards* $v_{P}$ *direction leads to the TNR increase, indicating the cutting-to-deformation wear ratio (CDR) increase.*

**Figure 5:**
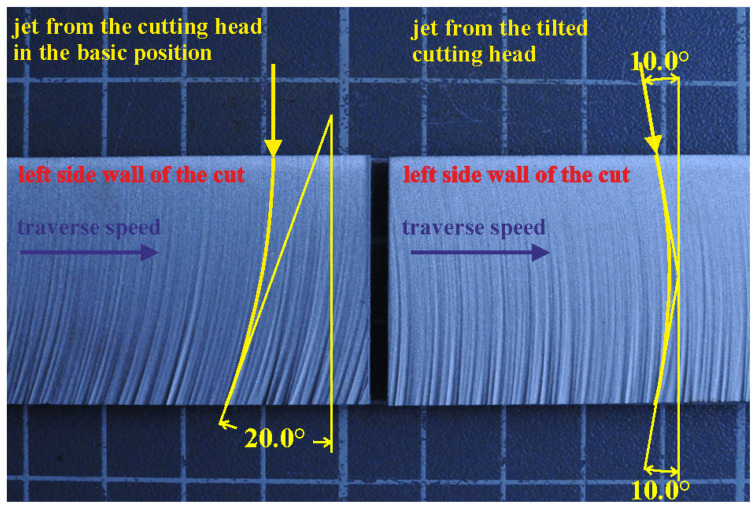
Example of striations for cutting with non-tilted and tilted jet.

Figure 5 shows the effect of the cutting head tilting towards the $v_{P}$ direction. It is claimed that the length of the jet trajectory with the appropriate conditions for cutting wear inside kerf increases up to a certain tilting angle.

Samples of the same dimensions prepared from the different metals were tested in experiments focused on the second and the third studied theorems.

### 4.1. Link between the TNR and the Traverse Speed Ratio

To study the first outlined theorem, the steel marked as 1.0038 in WRN standard (DIN RSt 37) was cut. The dimensions of all samples were 60 × 120 × T in mm, where T was the thickness. The thicknesses 10, 15, 20, 25 and 30 mm were cut in the first set of experiments, using a scale of the traverse speeds ($v_{P}$) starting from the 0.1 $v_{Plim}$ up to the 0.9 $v_{Plim}$, with the 0.2 $v_{Plim}$ steps. The cuts were 30 mm long. The tested theorem states that the TNR is identical for the identical actual-to-limit traverse speed ratios. The results are summarized in Table 3.

### 4.2. Link between the TNR and the Type of Wear

The second studied theorem, stating that the cutting and the deformation wear correspond to the TF and the NF respectively, is tested on metals used for other research purposes in the past [15,64]. All samples have identical dimensions: 60 × 120 × 10 in mm. The tested assumption is that the TNR decreases for higher $v_{P}$ values (in the case of the non-tilted cutting head), as the NF increases and the TF decreases. This phenomenon is accompanied by a worse quality, represented by the higher outlet declination angles. It is caused by the deformation wear increase with respect to the cutting one. The obtained results are summarized in Table 4.

The $v_{P}$ corresponding to the outlet declination angles 20° and 30° were used (see left part of Figure 5). Detailed introduction of the “declination angle” was presented in Reference [14]. The lower value of the declination angle is typical for the common economical commercial cutting (58 ± 1% of the $v_{Plim}$), and the higher one is usual for a low quality commercial cutting (76 ± 1% of the $v_{Plim}$). The respective $v_{P}$ values are calculated from Equation (5), which represents a modified version of Equation (11) from Reference [15], presented here as Equation (4):

(4) $$\theta =\theta_{lim}{\left(\frac{v_{P}}{v_{Plim}}\right)}^{\frac{3}{2}}$$

(5) $$v_{P}=v_{Plim}{\left(\frac{\theta }{\theta_{lim}}\right)}^{\frac{2}{3}}$$

The angle (θ) is the one expected at the bottom outlet edge when the impinging jet acts perpendicularly to the plan-parallel plate. The limit angle ($\theta_{lim}$) is equal to 45° within the jet effective working range when the jet energy is sufficient for both the cutting and the deformation wear. The limit traverse speed ($v_{Plim}$) is determined for each machine.

### 4.3. Link between the Cutting Head Tilting and the TNR/CDR

The third tested theorem is connected with the tilted cutting head. Two tilting angles (10° and 15°) are selected and tested on the different metal samples with dimensions 60 × 120 × 10 in mm. The angles values correspond to the values 20° and 30° of the non-tilted jets and, as mentioned above, these values are selected as the most typical ones respective to the traverse speeds used in the AWJ practice. Tilting angles correspond with the cutting head tilting used for a compensation of the respective trailback and the taper [39]. The results are summarized in Table 5 for these experiments.

The limit traverse speed can be either calculated from the theoretical model and the experimental results or it can be recalculated from the proven $v_{Plim}$ value known for settings at one machine to the value corresponding with the settings of another machine (e.g., using Equation (3) derived for recalculation of the $v_{Plim}$ between settings used in Ostrava and Milan, or any similar equation derived for a respective situation). The corresponding $v_{P}$ values for the declination angles on the outlet edge of the sample (20° and 30°) are calculated from Equation (5) for a jet impinging the material surface perpendicularly. The jet axis tilting angle (half of the expected bottom declination angle for the perpendicular jet impingement) is aimed at placement of the jet entry and exit points on the vertical line, normal to the material surface (Figure 5). The traverse speed for the tilting angle is identical as the traverse speed for the respective perpendicularly impacting jet. The cutting-wear zone is supposed to increase due to a longer path done at shallow impingement angles by the abrasive particles on the kerf front side, so the TNR ratio is higher for the inclined jets, namely for the higher traverse speed and the respective greater tilting angle.

## 5. Discussion of Results

The absolute values of the measured forces depend on the tested material and also on samples’ weights, the measuring device hysteresis, its fixing means and a position on the cutting device. The base frame of the force sensor has a limited stiffness. Therefore, the device position on the support, the grid stiffness on the residual jet energy attenuating vessel, the vibrations caused by the water churning in the vessel, the size and position of the weights used for fixing of the sensor and other mechanical factors can influence the absolute values of the measured forces in the separate time periods. However, results in Table 6 presenting data for two materials (steel and copper) measured in the different experiments show the consistency of the TNR data conclusively. The TNR remains unchanging under equivalent conditions, including the cut material properties and the abrasive waterjet characteristics. Therefore, the cases with the shifts in absolute values indicate a systematic affection induced in all axes simultaneously. Consequently, the force ratios can be used for studying the processes, as these ratios are much less influenced by the external factors than the absolute values themselves.

### 5.1. Discussion on the Link between the TNR and the Actual-to-Limit Traverse Speed Ratio

**Figure 6:**
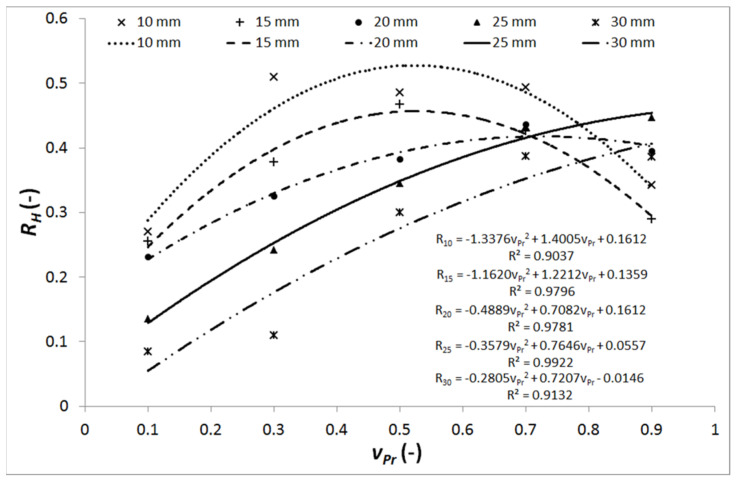
Tangential-to-normal (F_x_–F_z_) force ratio (*R_H_*) for the material thickness (*H*), depending on the $v_{Pr}$ (i.e., $v_{P}$-to-$v_{Plim}$ ratio): data from Table 3; the regression equations are calculated in Excel^®^.

Table 3 and the plot in Figure 6 show the relation between the TNR and the actual to limit traverse speed ratio ($v_{P}$-to-$v_{Plim}$ ratio) marked $v_{Pr}$. One of the most important effects on the result can be caused by energy exhausting of the jet-cutting of thick materials is impossible in the deformation wear mode, because slowed down and declined jet is unable to push the material out of the kerf. Therefore, only the cutting mode can be efficient in that case and it is closely tied up with the maximum (limit) declination angle. This limit decreases from the value 45° (relevant for cutting of the appropriate thicknesses to the jet energy) to the value around 22.5° (maximum for the cutting wear mode) [33]. Then, the TNR does not decrease with increasing traverse speed ratio $v_{Pr}$ up to the value one, because the $v_{Plim}$ covers only the cutting wear mode and the deformation one will never arise (see Figure 6). The limit traverse speed and the respective relative traverse speed are very important factors determining quality of the cutting walls, as it was investigated and confirmed by Sutowski et al. [65] and Sutowska et al. [66]. Their investigations were based on acoustic emissions [65] and deep studies of striations and roughness [66]. Combination of results in both publications conclusively support the general experience in the field of AWJ that relative traverse speed (as a portion of the limit one) directly determine the trailback, the taper, roughness, waviness and other qualitative characteristics of the cutting walls.

Increasing of the $v_{P}$ causes an increase of the deformation wear percentage but, simultaneously, the TF also increases, because of the slurry flow curvature. Then the slurry flow curvature reaches its limit and further TNR increase is impossible. Thinner samples, however, can be effectively penetrated in the deformation mode, provided that the jet energy is sufficient. The described situation results in the NF increase over the limit of balanced state observed for thicker samples (or less powerful pumps), i.e., the TNR starts decreasing for thinner samples at the $v_{P}$ higher than the half of the $v_{Plim}$ (see curves for 10 and 15 mm in Figure 6). The TNR decreases due to the NF increase during the slurry flow pushing through the cut material when the deformation wear can develop.

**Proof** **of** **Theorem** **1.** The results summarized in Table 3 and graphically presented in Figure 6 conclusively prove that the TNR is closely connected to the actual-to-limit traverse speed ratio marked $v_{Pr}$.

### 5.2. Discussion on the Link between the TNR and the CDR

**Figure 7:**
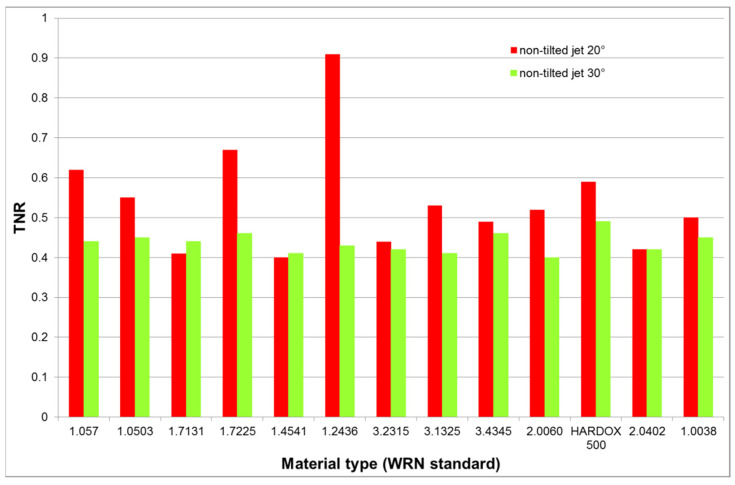
TNR for $v_{P}$ producing declination angle 20° (standard cutting quality) and 30° (low cutting quality).

Results reported in Table 4 show that the TNR decreases for increasing $v_{P}$ from the value producing the outlet declination angle of 20° to the one producing the angle of 30° at the perpendicular impact of the abrasive waterjet. It is evident from the lower TNR values for 30° compared to 20° graphically presented also in Figure 7. These results are also supporting the conclusions made in Section 5.1, i.e., for sufficient jet energy the increase of the traverse speed over a certain portion of the limit value (usually 0.5 $v_{Plim}$) causes decrease of the TNR. Since the cut material thickness is identical for all measured forces in Table 4 and the $v_{P}$ is set so that the declination angle at the jet outlet from the material is also identical, it is evident that the TNR is similar for both the wear resistant brittle steels and the plastic ductile metals like copper. These results are consistent with the phenomena described in previous discussion aimed at results presented in Table 3 and Figure 6. It is supposed that the TNR should be more or less identical for all the tested materials in each of the two pre-set experimental conditions (0.58 $v_{Plim}$ for 20° outlet declination angle and 0.76 $v_{Plim}$ for 30° outlet declination angle). Therefore, it is justified to calculate the average ratios for each of the selected traverse speeds. The average TNR determined for all measured materials decreases from 0.54 ± 0.13 (for the lower $v_{P}$) to 0.44 ± 0.02 (for the higher $v_{P}$). In other words, the average TNR measured for $v_{P}$ values producing declination angle 20° is 23% higher than the one measured for $v_{P}$ values respective to declination angle 30°. Selecting only steels, the respective values are 0.57 ± 0.16 and 0.44 ± 0.02, i.e., the average TNR decreases by 29.5% for the declination angle change from 20° to 30°. The respective values for tested non-steel metals, being 0.48 ± 0.04 and 0.42 ± 0.02, yield the average decrease only 14%. Using regression equation for mild steel presented in Figure 6 for 10 mm–thick sample, the respective values for angles 20° and 30° are 0.52 ± 0.07 and 0.45 ± 0.05, respectively, which means a decrease of 15.6%. These results confirm the statement that the TNR can be used as an indicator of the cutting quality. Decreasing of the TNR means increase of the deformation wear, i.e., lower cutting quality, for the traverse speeds higher than a half of the limit one.

**Proof** **of** **Theorem** **2.** The results of the force ratio measurements summarized in Table 4 and graphically presented in Figure 7 show that forces representing the interaction between the abrasive waterjet and the cut material (cutting–tangential one and deformation–normal one) indicate the prevailing type of material wear.

### 5.3. Discussion on the Link between the TNR and the CDR for Tilted Cutting Head

**Figure 8:**
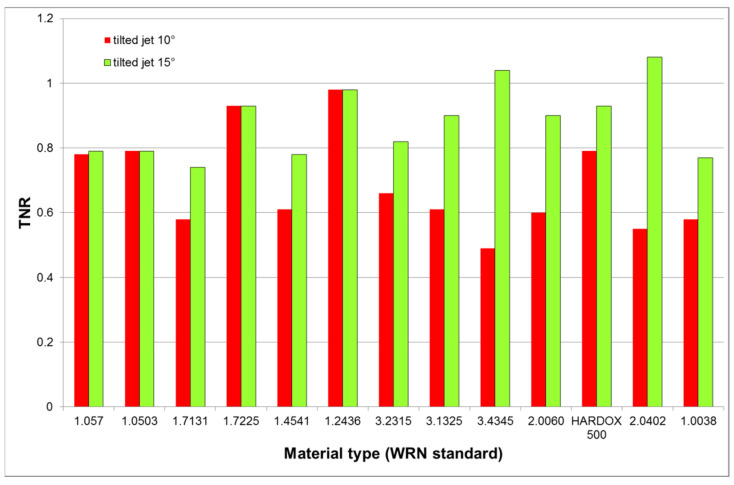
TNR for tilted jets (10° compensating deformation in the case of declination angle 20°; 15° compensating deformation in the case of declination angle 30°).

The results summarized in Table 5 show the TNR increase with the jet tilting conclusively, namely for the mild materials (see also the graphical presentation in Figure 8). The tilting angle increase from 10° to 15° intensifies the cutting wear for materials like aluminum alloys, copper or brass, therefore, the average value grows from 0.69 ± 0.14 to 0.88 ± 0.10, i.e., by 27.5%. It is given by the slurry flow curvature inside the produced kerf. The decomposition of the acting force into the TF and the NF components conclusively shows that the momentum variation increases in the $v_{P}$ direction.

**Figure 9:**
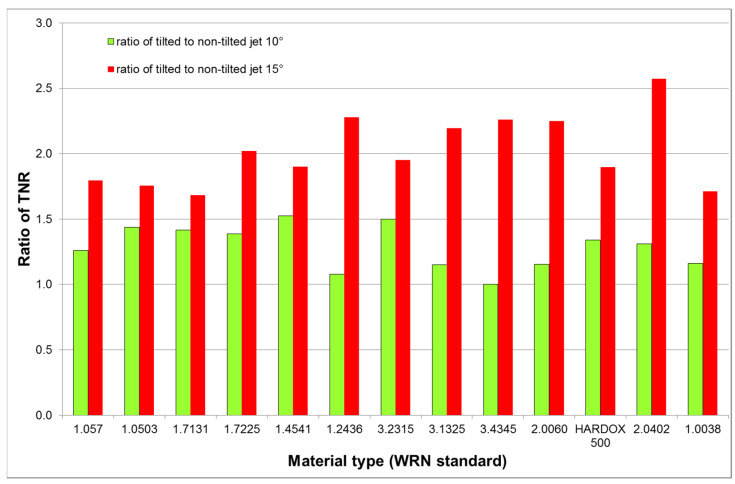
TNR comparison for tilted and non-tilted jets (10° tilting corresponds with 20° declination angle of non-tilted jet and 15° tilting corresponds to 30° of non-tilted jet).

The comparison of the tilted to the non-tilted jet at the equivalent conditions is presented in Figure 9. The average ratio of the tilted-to-non-tilted jet forces increased from the value 1.28 ± 0.16 for the declination angle 20° (10° jet compensation tilting) up to the value 2.02 ± 0.25 for the declination angle 30° (15° jet compensation tilting), i.e., by 57.8%. The results presented in Figure 9 are showing that tilting is much more efficient at higher traverse speeds assessed from the force decomposition point of view. However, the cutting wall quality need not be improved by a single tilting of the jet towards the direction of the traverse speed. Nevertheless, taking into account the inclination angle compensating the taper, the results may be much better. The linkage between the traverse speed and the taper was also investigated in past [37,38,39] and the presented models indicate that the appropriate compensation can be determined from the relative traverse speed, i.e., from the force measurements.

The results indicate that the more plastic materials, like aluminum alloys or copper and its alloys, have lower difference between the TNR measured for angles 20° and 30° for the perpendicular impact of the AWJ. However, tilting of the cutting head leads to a significant increase of the TNR for these materials. It indicates that the cutting head tilting improves the cutting-to-deformation wear ratio on these materials substantially.

**Proof** **of** **Theorem** **3.** The results of measurement with tilting cutting head summarized in Table 5 and graphically presented in Figure 8 and Figure 9 prove that tilting of the jet axis towards the $v_{P}$ direction leads to the TNR increase, indicating the cutting-to-deformation wear ratio (CDR) increase.

## 6. Conclusions

The force-measuring device intentionally developed for the force measurement in the AWJ cutting process is described and used for testing several theorems concerning the ratio of the tangential-to-normal force (TNR). Some new findings are presented. The TNR value reaches its maximum at around 50% of the limit traverse speed, $v_{Plim}$, for a medium sized material thickness when the AWJ energy is sufficient for development of both the cutting and the deformation wear in kerf. Increasing of the material thickness induces shifting of the maximum TNR towards the limit traverse speed, $v_{Plim}$, because the depth produced in the deformation wear decreases to zero. The average TNR values can reflect the cutting-to-deformation wear ratio (CDR). The experimental results also show the following:

- The CDR decreases by increasing the $v_{P}$ (which means increasing the declination angle at the kerf bottom);
- The jet axis tilting towards the $v_{P}$ direction induces a TNR increase being more extent for less brittle metals like aluminum, copper and their alloys;
- The tilting angle increase from 10° to 15° changes the average TNR from 0.69 ± 0.14 to 0.88 ± 0.10, i.e., by 27.5%;
- As the $v_{P}$ increases from the value producing the bottom declination angle of 20° to the value producing the bottom declination angle of 30°, together with their respective cutting head tilting angles of 10° and 15°, the tilted-to-non-tilted force ratio increases from 1.28 ± 0.16 to 2.02 ± 0.25, i.e., by 57.8%.

The force behavior indicates an increase of the CDR for the tilted jets. Testing of this conclusion is planned on other materials (rocks, plastics and glass) and for the different waterjet generating pressures or other conditions.

## Figures and Tables

**Table 1:** Values of preset cutting parameters.

Variable (Unit)	Ostrava (PTV WJ 1020-1Z-EKO)	Milano (Tecnocut IDRO 1740) (Intermac Primus 322)
Water pressure in pump (MPa)	380	350
Water nozzle (orifice) diameter (mm)	0.254	0.330
Focusing tube diameter (mm)	1.02	1.02
Focusing tube length (mm)	76	76
Abrasive mass flow (g/min)	250	300
Mean abrasive grain size ^(*)^ (mm)	0.25	0.25
Abrasive type ^(^**^)^	AG	AG
Stand-off distance (mm)	2	3

^(*)^ Measurements of AG garnet 80 mesh is performed at the certified Laboratory of Powdery Materials at the VŠB-Technical University of Ostrava on the machine Mastersizer 2000 and confirmed on the Fritsch Analysette 22 MicroTec plus. ^(^**^)^ Abrasive type is almandine Australian Garnet (AG).

**Table 2:** Traverse speed, $v_{Plim}$, determined for cutting of samples 10 mm thick, using equipment in Ostrava and Milano.

Marking of Samples	$v_{PlimO}$	$v_{PlimM}$
WRN (DIN) Standard	mm/min	mm/min
1.057 (St 52-3) steel	250	325
1.0503 (C 45) steel	200	260
1.7131 (16 MnCr 5) steel	220	286
1.7225 (42 CrMo 4) steel	180	234
1.4541 (X6 CrNiTi 18 10) steel	200	260
1.2436 (X210 CrW 12) steel	160	208
3.2315 (AlMgSi1Mn) duralumin	750	975
3.1325 (AlCu4MgSi) duralumin	760	988
3.4345 (AlZn5Mg3Cu) duralumin	720	936
2.0060 (E-Cu57) copper	380	494
HARDOX 500 PLATE steel	135	176
2.0402 (CuZn40Pb2) brass	350	455
1.0038 (RSt 37-2) steel	246	320

**Table 3:** TNR measured with respect to relative traverse speed, $v_{Pr}$ (a ratio of $v_{Plim}$).

Material Type	Thickness	$v_{PlimM}$ ^(*)^	Relative Traverse Speed $v_{Pr}$				
WRN (DIN) Standard	mm	mm/min	0.1	0.3	0.5	0.7	0.9
1.0038 (RSt 37)	10	320	0.271	0.510	0.486	0.493	0.342
1.0038 (RSt 37)	15	220	0.255	0.377	0.468	0.426	0.290
1.0038 (RSt 37)	20	150	0.231	0.325	0.383	0.436	0.395
1.0038 (RSt 37)	25	102	0.135	0.242	0.344	0.433	0.446
1.0038 (RSt 37)	30	72	0.084	0.110	0.300	0.387	0.386

^(*)^ $v_{Plim}$ determined for experimental settings used in Milano.

**Table 4:** TNR ratio on 10 mm–thick metal samples.

Material Type	Declination Angle 20°		Declination Angle 30°		Ratio for
	$v_{P}$	TNR	$v_{P}$	TNR	20°/30°
WRN (DIN) Standard	mm/min	NT-Head ^(*)^	mm/min	NT-Head ^(*)^	Forces Ratios
1.057 (St 52-3)	189	**0.62**	248	**0.44**	***1.43***
1.0503 (C 45)	151	**0.55**	198	**0.45**	***1.22***
1.7131 (16 MnCr 5)	167	**0.41**	218	**0.44**	***0.95***
1.7225 (42 CrMo 4)	136	**0.67**	179	**0.46**	***1.46***
1.4541 (X6 CrNiTi 18 10)	151	**0.40**	198	**0.41**	***0.96***
1.2436 (X210 CrW 12)	121	**0.91**	159	**0.43**	***2.10***
3.2315 (AlMgSi1Mn)	568	**0.44**	744	**0.42**	***1.03***
3.1325 (AlCu4MgSi)	575	**0.53**	754	**0.41**	***1.29***
3.4345 (AlZn5Mg3Cu)	545	**0.49**	714	**0.46**	***1.05***
2.0060 (E-Cu57)	288	**0.52**	377	**0.40**	***1.28***
HARDOX 500 PLATE	102	**0.59**	134	**0.49**	***1.21***
2.0402 (CuZn40Pb2)	265	**0.42**	347	**0.42**	***1.01***
1.0038 (RSt 37-2)	186	**0.50**	244	**0.45**	***1.13***

^(*)^ NT-head, non-tilted cutting head.

**Table 5:** Tangential-to-normal force ratio measured for tilted AWJ cutting.

	Tilting Angle 10°		Ratio of	Tilting Angle 15°		Ratio of
Material Type	$v_{P}\;$	TNR	T/NT	$v_{P}\;$	TNR	T/NT
WRN (DIN) Standard	mm/min	T-Head ^(*)^	Forces	mm/min	T-Head ^(*)^	Forces
1.057 (St 52-3)	189	**0.78**	***1.25***	248	**0.79**	***1.82***
1.0503 (C 45)	151	**0.79**	***1.43***	198	**0.79**	***1.76***
1.7131 (16 MnCr 5)	167	**0.58**	***1.41***	218	**0.74**	***1.71***
1.7225 (42 CrMo 4)	136	**0.93**	***1.38***	179	**0.93**	***2.01***
1.4541 (X6 CrNiTi 18 10)	151	**0.61**	***1.53***	198	**0.78**	***1.87***
1.2436 (X210 CrW 12)	121	**0.98**	***1.08***	159	**0.98**	***2.26***
3.2315 (AlMgSi1Mn)	568	**0.66**	***1.52***	744	**0.82**	***1.93***
3.1325 (AlCu4MgSi)	575	**0.61**	***1.15***	754	**0.90**	***2.18***
3.4345 (AlZn5Mg3Cu)	545	**0.49**	***1.01***	714	**1.04**	***2.26***
2.0060 (E-Cu57)	288	**0.60**	***1.15***	377	**0.90**	***2.22***
HARDOX 500 PLATE	102	**0.79**	***1.33***	134	**0.93**	***1.92***
2.0402 (CuZn40Pb2)	265	**0.55**	***1.30***	347	**1.08**	***2.60***
1.0038 (RSt 37-2)	186	**0.58**	***1.16***	244	**0.77**	***1.73***

^(*)^ T-head, tilted cutting head; T/NT, ratio of forces determined for tilted and non-tilted cutting head.

**Table 6:** Absolute values of TF, NF and the respective TNR for measurements performed at the identical conditions on two selected materials in various days—the only difference is changed position and fixing of the sensor, because of other works performed on the cutting device in the meantime.

Material	Non-Tilted 20°			Tilted 20°			Non-Tilted 30°			Tilted 30°		
	TF	NF	TNR	TF	NF	TNR	TF	NF	TNR	TF	NF	TNR
	(N)	(N)		(N)	(N)		(N)	(N)		(N)	(N)	
Steel 1.0038 (RSt37) 10 mm	1.64	3.63	**0.45**	1.24	2.10	**0.59**	2.42	5.71	**0.42**	2.06	2.34	**0.88**
	1.31	2.38	**0.55**	1.37	2.59	**0.53**	2.03	4.25	**0.48**	2.02	2.47	**0.82**
	1.17	2.39	**0.49**	1.35	2.17	**0.62**	1.74	3.82	**0.46**	2.58	3.49	**0.74**
	2.84	5.61	**0.51**	2.79	4.60	**0.61**	-	-	**-**	-	-	**-**
**Average**			**0.50 ± 0.04**			**0.59 ± 0.03**			**0.45 ± 0.02**			**0.81 ± 0.06**
Copper 2.0060 (E-Cu57) 10 mm	4.89	9.41	**0.52**	3.03	5.08	**0.60**	3.90	9.62	**0.40**	4.88	5.44	**0.90**
	1.44	3.47	**0.42**	1.34	2.15	**0.62**	2.27	5.57	**0.41**	2.84	3.16	**0.90**
	1.05	2.33	**0.45**	1.28	1.74	**0.74**	1.75	3.74	**0.47**	2.67	3.91	**0.68**
	1.05	2.35	**0.45**	1.37	2.07	**0.66**	1.48	3.77	**0.39**	2.30	2.00	**1.15**
**Average**			**0.46 ± 0.04**			**0.66 ± 0.05**			**0.42 ± 0.03**			**0.91 ± 0.17**

## Data Availability

No publicly archived datasets are reported or used.
